# Hereditary 1,25-dihydroxyvitamin D-resistant rickets (HVDRR) caused by a *VDR* mutation: A novel mechanism of dominant inheritance

**DOI:** 10.1016/j.bonr.2015.05.001

**Published:** 2015-05-07

**Authors:** Tsuyoshi Isojima, Michiyasu Ishizawa, Kazuko Yoshimura, Mayuko Tamura, Shinichi Hirose, Makoto Makishima, Sachiko Kitanaka

**Affiliations:** a Department of Pediatrics, Graduate School of Medicine, The University of Tokyo, 7-3-1 Hongo, Bunkyo-ku, Tokyo 113-8655, Japan; b Division of Biochemistry, Department of Biochemical Sciences, Nihon University School of Medicine, 30-1 Oyaguchi-kamicho, Itabashi-ku, Tokyo 173-8610, Japan; c Department of Pediatrics, School of Medicine, Fukuoka University, 7-45-1 Nanakuma, Jonan-ku, Fukuoka 814-0180, Japan

**Keywords:** Hereditary vitamin D resistant rickets, Dominant negative effect, Vitamin D receptor

## Abstract

Hereditary 1,25-dihydroxyvitamin D-resistant rickets (HVDRR) is caused by mutations in the *VDR* gene, and its inheritance is autosomal recessive. In this report, we aimed to confirm whether HVDRR is occasionally inherited as a dominant trait. An 18-month-old Japanese boy was evaluated for short stature and bowlegs. His father had been treated for rickets during childhood, and his paternal grandfather had bowlegs. We diagnosed him with HVDRR based on laboratory data and radiographic evidence of rickets. Sequence analyses of *VDR* were performed, and the functional consequences of the detected mutations were analyzed for transcriptional activity, ligand binding, and interaction with the retinoid X receptor, cofactors, and the vitamin D response element (VDRE). A novel mutation (Q400LfsX7) and a reported variant (R370H) were identified in the patient. Heterozygous Q400LfsX7 was detected in his father, and heterozygous R370H was detected in his healthy mother. Functional studies revealed that the transcriptional activity of Q400LfsX7-VDR was markedly disturbed. The mutant had a dominant-negative effect on wild-type-VDR, and the ligand binding affinity of Q400LfsX7-VDR was completely impaired. Interestingly, Q400LfsX7-VDR had a strong interaction with corepressor NCoR and could interact with VDRE without the ligand. R370H-VDR was functionally similar to wild-type-VDR. In conclusion, we found a dominant-negative mutant of VDR causing dominantly inherited HVDRR through a constitutive corepressor interaction, a mechanism similar to that in dominantly inherited thyroid hormone receptor mutations. Our report together with a reported pedigree suggested a distinct inheritance of HVDRR and enriched our understanding of VDR abnormalities.

## Highlights

• We report the pedigree of dominantly inherited hereditary vitamin D resistant rickets.
• A novel *VDR* mutation (Q400LfsX7) was identified in the pedigree.
• The mutant had a dominant-negative effect on the wild type VDR and interacted strongly with corepressor NCoR.
• The mechanism for dominantly inheritance was similar to that in dominantly inherited thyroid hormone receptor mutations.

## 1 Introduction

The hormonal metabolite of vitamin D, 1,25-dihydroxyvitamin D [1,25(OH)_2_D_3_] regulates calcium homeostasis, cellular differentiation, and immune function through its binding to the vitamin D receptor (VDR), a transcription factor belonging to the steroid/nuclear receptor superfamily (Feldman and Malloy, 2014, Haussler et al., 2013, Rachez and Freedman, 2000, Malloy et al., 2011). The regulation of specific gene transcription by VDR requires it to form a heterodimer with the retinoid X receptor (RXR); this heterodimer then binds to vitamin D response elements (VDREs) in the promoter region of target genes, releasing corepressor proteins and recruiting coactivator proteins. 1,25(OH)_2_D_3_ binding to VDR causes the repositioning of helix H12, which contains an activation function 2 (AF-2) domain, allowing for the recruitment of coactivators (Rochel et al., 2000).

Hereditary 1,25-dihydroxyvitamin D-resistant rickets (HVDRR) (OMIM #277440), also known as vitamin D-resistant rickets type 2A, is a rare monogenic disorder caused by mutations in the *VDR* gene (Malloy and Feldman, 2010). Patients with HVDRR often have consanguinity in the family and display a number of clinical features including early onset rickets, hypocalcemia, and secondary hyperparathyroidism. Moreover, they have significantly elevated serum 1,25(OH)_2_D levels; this differentiates this condition from 1α-hydroxylase deficiency, also known as vitamin D-dependent rickets type 1A (Kitanaka et al., 1998). Multiple mutations in the *VDR* gene have been reported to cause HVDRR (Malloy et al., 2014). Mutations in the DNA binding domain (DBD) of the *VDR* gene interfere with VDR–DNA interactions and result in the loss of VDR function, and these are usually associated with alopecia (Malloy et al., 2014). Mutations in the *VDR* ligand binding domain (LBD) alter the ligand binding affinity in various ways. These LBD mutations can result in partial or total hormone unresponsiveness and can be associated with alopecia (Malloy et al., 2002).

Patients with HVDRR usually have biallelic mutations in the *VDR* gene (Feldman and Malloy, 2014). Those with heterozygous mutations (i.e., the parents of sufferers) show no symptoms and have normal bone development (Malloy et al., 2004, Malloy et al., 2011). Recently, a single family has been reported to demonstrate dominant inheritance caused by a *VDR* mutation with a dominant-negative effect (Malloy et al., 2011). Here, we report a family with HVDRR showing dominant inheritance, in which we identified a novel mutation with a dominant-negative effect and enhanced corepressor interaction. This is the first report of a dominant-negative VDR mutation demonstrating a constitutive corepressor interaction, a mechanism similar to that in dominantly inherited thyroid hormone receptor (TR) mutations (Safer et al., 1998, Liu et al., 1998, Fozzatti et al., 2011, Fozzatti et al., 2013, Bochukova et al., 2012).

## 2 Subjects and methods

### 2.1 Patient clinical observation

An 18-month-old Japanese boy was referred to our institute and evaluated for short stature and bowlegs. His body length and weight were 80.2 cm [− 2.0 standard deviations (SD)] and 8.6 kg (− 1.6 SD), respectively. He is the first child of nonconsanguineous parents, and his birth history was unremarkable. He started walking at the age of 14 months, but his parents felt he had a slight waddling gait. Intellectual development was within normal limits. His father had been treated for a short period with some vitamin D for rickets during childhood, which was considered to be due to simple vitamin D deficiency; his height was 173 cm, and he had a slight bowing of his legs. The patient's paternal grandfather also had bowlegs without alopecia, although he had never been treated.

**Fig. 1:**
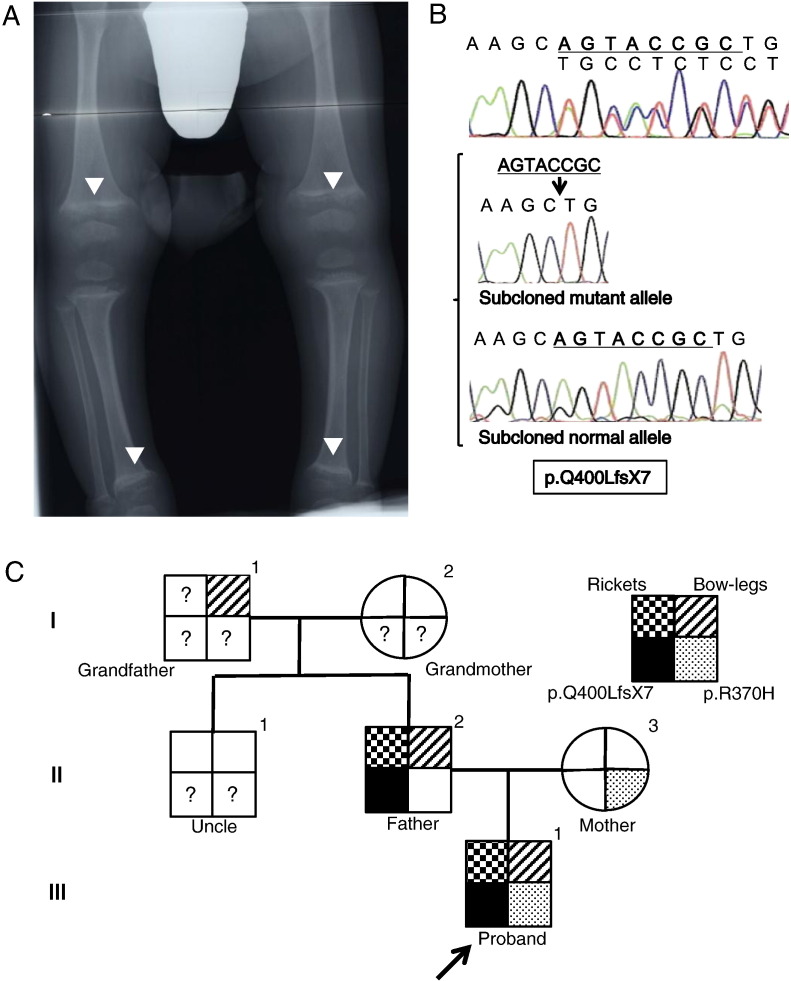
Radiograph of the patient, a chromatogram of the novel mutation, and the family tree. (A) Radiograph of the patient at diagnosis. It showed cupping, fraying and flaring indicating that the patient had evident rickets. (B) The novel p.Q400LfsX7 mutation. In the chromatogram: nucleotides in bold black letters with underline indicate an 8-base pair deletion; and the lower part shows the subcloned normal and mutant sequences. (C) The pedigree analysis in this study.

Radiographic study of the hands and knees was consistent with rickets (Fig. 1A). Blood biochemistry showed normal serum calcium (Ca) (2.2 mmol/l; reference range, 2.1–2.4) and 25(OH)D levels (20 ng/ml; insufficiency, < 20). However, they showed slightly decreased serum phosphorus (P) (1.1 mmol/l; reference range, 1.3–2.0) and elevated serum alkaline phosphatase (ALP) (3346 U/l; reference range, 171–785), intact parathyroid hormone (iPTH) (480 pg/ml; reference range, 10–65), and 1,25(OH)_2_D levels (304 pg/ml; reference range, 20–70). On the basis of these findings of obvious rickets, with elevated 1,25(OH)_2_D, ALP, and iPTH levels and a normal 25(OH)D level, we diagnosed him with HVDRR. However, we could not exclude the possibility of vitamin D deficiency. He was effectively treated with 2 μg alfacalcidol daily without any calcium supplements, and his rickets, ALP, iPTH levels improved. The dose was tapered and stopped at the age of 4 years. At the last follow-up (age, 5 years and 11 months), he had neither rickets nor alopecia and measured 106.8 cm (− 1.3 SD) in height. His blood biochemistry showed normal Ca (2.4 mmol/l), P (1.5 mmol/l), ALP (622 U/l), and iPTH (53 pg/ml), but elevated 1,25(OH)_2_D (271 pg/ml) levels.

### 2.2 *VDR* gene analysis

We obtained informed consent for DNA analysis from the parents, and the Ethics Committee of The University of Tokyo approved the study. Genomic DNA was extracted from peripheral white blood cells using a QIAamp DNA Blood Midi Kit (Qiagen, Hilden, Germany). The entire coding region and exon–intron boundaries of the *VDR* gene were amplified from genomic DNA by polymerase chain reaction (PCR) using the designed PCR primers. The details of the primers and the PCR conditions will be provided on request. Subsequently, PCR products were sequenced using an ABI Prism BigDye Terminator Cycle Sequencing Ready Reaction Kit (PE Applied Biosystems, Foster City, CA) and the forward and reverse primers from the PCR amplification. Direct sequencing in both directions was performed on an autosequencer (ABI PRISM 310, Genetic Analyzer; Applied Biosystems). Detected mutations were confirmed by cloning the PCR products into the pCR 2.1 vector using a TOPO TA Cloning kit (Invitrogen, Carlsbad, CA, U.S.A.). The clones derived from both alleles were sequenced.

### 2.3 Construction of plasmids

Expression plasmids for wild-type full-length human VDR or RXRα and a reporter plasmid for human 24-hydroxylase promoter (− 367 to 0) in pGL3 were provided by S. Kato. The expression plasmids hVDR-pCMX, hVDR-flag-pCMX, hVDR-VP16-pCMX, RXRα-GAL4-pCMX, GRIP1-GAL4-pCMX, and NCoR-GAL4-pCMX have been reported previously (Inaba et al., 2007). GAL4-responsive MH100(UAS)x4-tk-LUC and VDR-responsive Sppx3-tk-LUC were also used in the luciferase reporter assay (Nakano et al., 2005, Igarashi et al., 2007). The mutant plasmids (Q400LfsX7 and R370H) were created with a Quick Change Site-directed mutagenesis kit (Stratagene, La Jolla, CA) according to the manufacturer's protocol (Sato et al., 2005). *VDR* mutant constructs were sequence-verified to have no extra mutations.

### 2.4 Transcriptional activity

COS-1 cells were cultured in Dulbecco's modified Eagle's medium (DMEM) supplemented with 10% fetal bovine serum, 100 unit/ml penicillin, and 100 μg/ml streptomycin at 37 °C in a humidified atmosphere containing 5% CO_2_. Transfections in COS-1 cells were performed by modification of previously reported methods (Sato et al., 2005, Jurutka et al., 2000). Cells that were cultured in 24-well plates were transfected with 800 ng DNA, including 300 ng 24-hydroxylase-pGL3 promoter luciferase, the indicated amounts of each human VDR expression plasmids, and 1 ng pRL-CMV (Promega, Madison, WI) using Lipofectamine 2000 (Invitrogen, Carlsbad, CA). Four hours after transfection, the indicated amounts of ligand were added. After 24 h, transcriptional activity was assayed using a Dual-Luciferase Reporter Assay System (Promega). The luciferase activities of human 24-hydroxylase promoter luciferase plasmid were normalized to the luciferase activities of pRL-CMV. Transient transfections were performed in triplicate, and each experiment was repeated at least three times.

### 2.5 Mammalian two-hybrid analyses

Human embryonic kidney (HEK) 293 cells were cultured in DMEM containing 5% fetal bovine serum and an antibiotic–antimycotic (Nacalai, Kyoto) at 37 °C in a humidified atmosphere containing 5% CO_2_. Transfections in HEK293 cells were performed by the calcium phosphate coprecipitation analysis as described previously (Adachi et al., 2005). Cells that were cultured in 96-well plates were transfected with 50 ng GAL4-responsive MH100(UAS)x4-tk-LUC reporter plasmid or VDR-responsive Sppx3-tk-LUC, 20 ng β-galactosidase-pCMX, 15 ng of each VDR, and/or the cofactor expression plasmid and pGEM carrier DNA for a total 150 ng DNA. Eight hours after transfection, the indicated amounts of ligands were added. Cells were harvested approximately 16–24 h after treatment, and luciferase and β-galactosidase activities were assayed using a luminometer and a microplate reader (Molecular Devices, Sunnyvale, CA). Luciferase data were normalized to an internal β-galactosidase control and were represented as the mean (± SD) of triplicate assays.

### 2.6 Competitive ligand-binding assay

LBDs of human *VDR* and its mutants were cloned into the GST-fusion vector pGEX-4T1 (Amersham Pharmacia Biotech, Piscataway, NJ). GST–VDR fusion proteins were expressed in BL21 DE3 cells (Promega) and purified with glutathione sepharose beads (Amersham Pharmacia Biotech). A competitive ligand-binding assay was performed by modification of previously reported methods (Nakajima et al., 1994, Solomon et al., 2001). Briefly, 500 ng GST fusion proteins were bound to glutathione sepharose and incubated with [26,27-methyl-^3^H]1α,25(OH)_2_D_3_ (Amersham Pharmacia Biotech) in the presence or absence of the nonradioactive ligand in a buffer (10 mM Tris–HCl, pH 7.6; 1 mM EDTA; 300 mM KCl; 1 mM dithiothreitol; 10% glycerol) for 3 h at 4 °C. After washing twice, the protein and bound 1α,25(OH)_2_D_3_ were resuspended in 200 μl of the binding buffer, and a 150 μl sample was assessed by liquid scintillation counting.

## 3 Results

### 3.1 Mutation analysis of the *VDR* gene

The genomic analyses for the *VDR* gene revealed that the patient had a novel deletion mutation and a reported variant (rs202139940). The mutation is an 8-base pair deletion in exon 10 of the *VDR* gene, c.1199–1206 del, p.Q400LfsX7, which is predicted to result in a frameshift from codon 400 and premature termination just before helix H12 (Fig. 1B). This detected mutation was heterozygous in his father. The variant (rs202139940) is a G > A transition in exon 10 of the *VDR* gene, which is predicted to result in a substitution of arginine to histidine in codon 370 (R370H). This variant was also found to be heterozygous in his healthy mother. Both the patient and his mother were heterozygous for the *FokI* polymorphism (F/f) that alters the translational site from M1 to M4 (Jurutka et al., 2000, Haussler et al., 1998), while his father was homozygous for the f/M1 alleles.

### 3.2 Transcriptional activity of Q400LfsX7-VDR and R370H-VDR

**Fig. 2:**
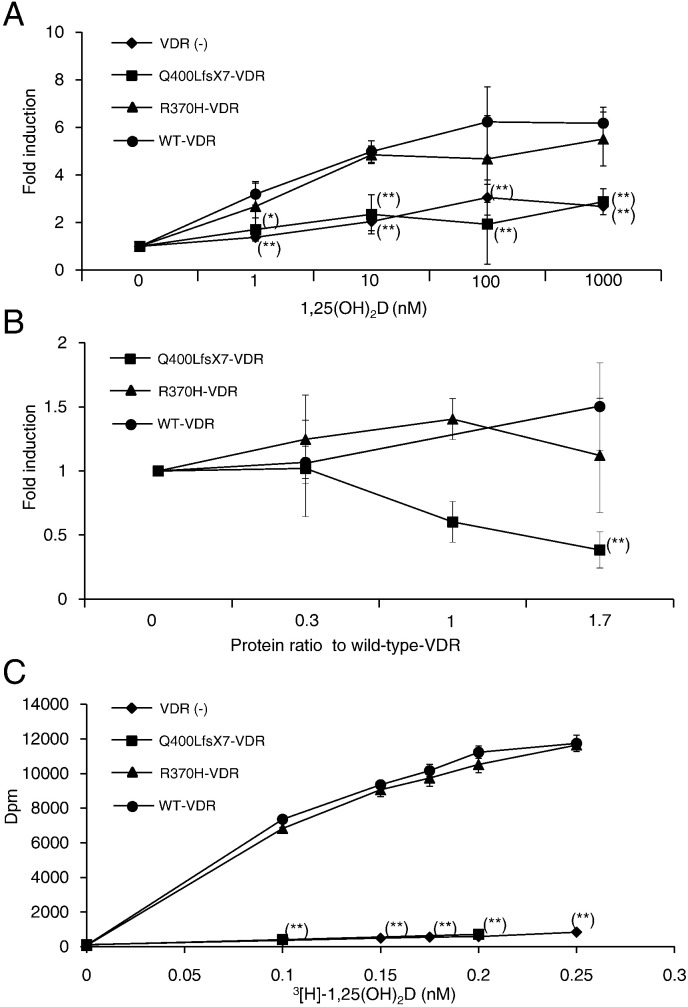
Functional analysis of the VDR mutants. (Luciferase activity of the reporter is shown as relative light unit compared to that in cells transfected with control vector and treated with vehicle control. Error bars represent one standard deviation. (*) and (**) denote the statistically significant difference comparing to wild-type VDR at p < 0.05 and p < 0.01, respectively). (A) The transcriptional activity of Q400LfsX7 or R370H-VDR in COS-1 transfected cells. (B) Analysis of the dominant-negative effect of Q400LfsX7-VDR on wild-type-VDR transcriptional activity in COS-1 transfected cells with 5 nM of 1,25(OH)_2_D_3_. (C) Direct binding of 1,25(OH)_2_D_3_ to VDR. GST–VDR fusion proteins or GST control proteins incubated with increasing concentrations of [^3^H]1,25(OH)_2_D_3_ in the presence or absence of 400-fold excess nonradioactive 1,25(OH)_2_D_3_.

To examine whether the detected Q400LfsX7 mutation and the R370H variant can affect VDR transactivation, wild-type Q400LfsX7-VDR and R370H-VDR were transiently overexpressed in COS-1 cells, and transcriptional activity was analyzed using the reporter 24-hydroxylase promoter. R370H-VDR showed similar transcriptional activity to that of wild-type-VDR (Fig. 2A). However, Q400LfsX7-VDR had negligible transcriptional activity. Similar results were also observed in HEK293 cells (data not shown). These results indicated that the Q400LfsX7-VDR mutant had completely lost all transcriptional activities, whereas R370H remained normal.

### 3.3 The dominant-negative effect of Q400LfsX7-VDR on wild-type VDR

We hypothesize that the heterozygous Q400LfsX7 mutation was the molecular cause of his HVDRR. We examined the dominant-negative effect of Q400LfsX7-VDR on the wild-type product by increasing the mutant-to-wild-type protein ratio with 5 nM of 1,25(OH)_2_D_3_. When equal amounts of wild-type VDR and Q400LfsX7-VDR proteins were expressed, transcriptional activity was reduced by approximately 50%, and the increasing amounts of the mutant VDR further repressed wild-type VDR transcriptional activity (Fig. 2B). This finding demonstrated that Q400LfsX7-VDR had a dominant-negative effect on wild-type VDR. However, R370H-VDR did not affect wild-type VDR transcriptional activity (Fig. 2B). Moreover, Q400LfsX7-VDR showed a dominant-negative effect on R370H-VDR activity, similar to its effect on wild-type-VDR (data not shown). Similar results were observed in HEK293 cells using VDR-responsive Sppx3-tk-LUC (data not shown).

### 3.4 Ligand binding ability of Q400LfsX7-VDR and R370H-VDR

The binding affinity of 1,25(OH)_2_D_3_ of Q400LfsX7 or R370H-VDR was examined by the competitive binding assay. Isotopically labeled 1,25(OH)_2_D_3_ was incubated with glutathione-S-transferase (GST)–VDR proteins in the presence or absence of excess unlabeled 1,25(OH)_2_D_3_, and specific binding of 1,25(OH)_2_D_3_ was calculated. [^3^H]1,25(OH)_2_D_3_ effectively bound to wild-type-VDR and R370H-VDR but did not bind to Q400LfsX7-VDR. This result indicated that ligand binding affinity of Q400LfsX7-VDR was completely impaired (Fig. 2C).

### 3.5 Interaction with RXR, coactivator, and corepressor of Q400LfsX7-VDR and R370H-VDR

**Fig. 3:**
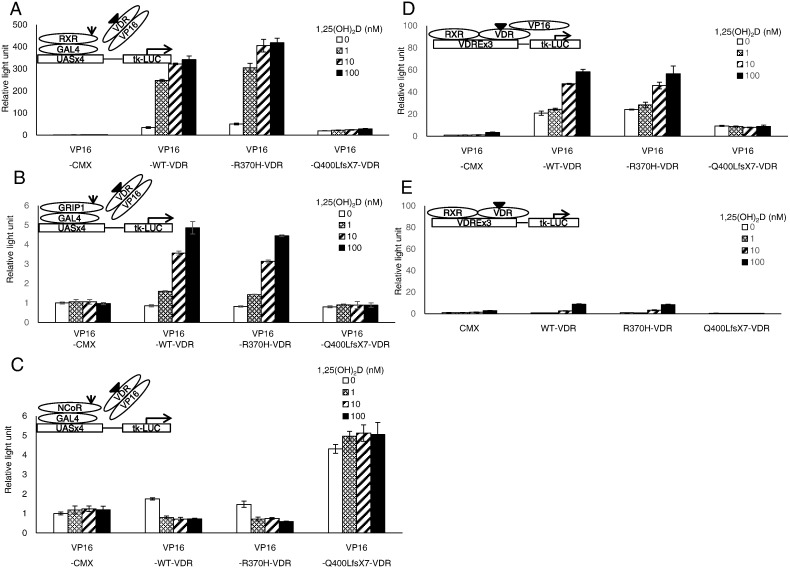
RXR, coactivator, corepressor, and VDRE interactions with Q400LfsX7-VDR or R370H-VDR in HEK293 transfected cells. (Luciferase activity of the reporter is shown as relative light unit compared to that in cells transfected with control vector and treated with vehicle control. Error bars represent one standard deviation). (A) RXR interactions with Q400LfsX7-VDR or R370H-VDR. Mammalian two-hybrid analysis using hVDR-VP16-pCMX and RXRα-GAL4-pCMX in HEK293 cells. (B) Coactivator GRIP1 interactions with Q400LfsX7-VDR or R370H-VDR. Mammalian two-hybrid analysis using hVDR-VP16-pCMX and GRIP1-GAL4-pCMX in HEK293 cells. (C) Corepressor NCoR interactions with Q400LfsX7-VDR or R370H-VDR. Mammalian two-hybrid analysis using hVDR-VP16-pCMX and NCoR-GAL4-pCMX in HEK293 cells. (D) VDRE interactions with Q400LfsX7-VDR or R370H-VDR. VP16-VDR lanes. (E) VDRE interactions with Q400LfsX7-VDR or R370H-VDR. VDR lanes. Transcriptional activity of Q400LfsX7-VDR was augmented with a VP16 chimeric receptor in a ligand-independent manner [compare VP16-VDR lanes (panel D) to VDR lanes (panel E)].

Interactions between VDR mutants, RXR, the coactivator, or the corepressor were analyzed by a mammalian two-hybrid assay using hVDR-VP16-pCMX, RXRα-GAL4-pCMX, GRIP1-GAL4-pCMX, and NCoR-GAL4-pCMX, respectively. HEK293 cells were cotransfected with hVDR-VP16-pCMX, the MH100(UAS)x4-tk-LUC reporter, and RXRα-GAL4-pCMX and were treated with increasing concentrations of 1,25(OH)_2_D_3_. R370H-VDR interacted with RXRα, similar to wild-type-VDR, in a dose-dependent manner of the ligand. However, Q400LfsX7-VDR did not interact with RXRα (Fig. 3A). This finding suggested that Q400LfsX-VDR had impaired heterodimerization with RXR. Next, we analyzed the interaction with a coactivator, GRIP1 (Fig. 3B). 1,25(OH)_2_D_3_ induced concentration-dependent associations of GRIP1 with wild-type or R370H-VDR. On the other hand, the association of Q400LfsX7-VDR with GRIP1 was not identified in any amounts of 1,25(OH)_2_D_3_ (Fig. 3B). Then, the interaction of VDR with a corepressor, NCoR, was evaluated with similar experiments (Fig. 3C). Wild-type or R370H-VDR showed an interaction with NCoR in the absence of the ligand, which decreased by adding 1,25(OH)_2_D_3_. In contrast, Q400LfsX7-VDR had a strong interaction with NCoR, which did not decrease by adding 1,25(OH)_2_D_3_ (Fig. 3C). These data indicated that Q400LfsX7 displayed a defective release of NCoR in response to the ligand.

### 3.6 VDRE interactions with Q400LfsX7-VDR or R370H-VDR

We next assessed the interaction of the VDR mutants and VDRE by transfecting VP16-VDR chimeric receptors together with the VDRE reporter. Because of ligand-independent activity, the luciferase activities of VP16 chimeric receptors showed an interaction between the receptor and the binding element (Makishima et al., 2002, Adachi et al., 2004, Endo-Umeda et al., 2012). HEK293 cells were transfected with VP16-VDR chimeric mutants together with the luciferase reporter containing a VDR-responsive everted repeat-6 element from the CYP3A4 promoter (Makishima et al., 2002). The activity was compared with the activity of those without VP16 to differentiate from transcriptional activity. Wild-type VP16-VDR or VP16-R370H-VDR induced luciferase activities in the absence of the ligand, and adding the ligand increased the activity (compare Fig. 3D lanes 5–12 with Fig. 3E lanes 5–12). This result suggested that these VDRs interacted with VDRE without the ligand and had ligand-dependent enhancement. In contrast, VP16-Q400LfsX7-VDR had ligand-independent activity (compare Fig. 3D lanes 13–16 with Fig. 3E lanes 13–16). Thus, Q400LfsX7-VDR could interact with VDRE, although the interaction was not influenced by the ligand (Fig. 3D lanes 13–16).

## 4 Discussion

We have identified and presented a novel *VDR* mutation (Q400LfsX7) with a dominant negative effect on wild-type-VDR in a family with dominantly inherited HVDRR. Because HVDRR is usually transmitted autosomal recessively, and because our patient had the R370H variant, we initially believed that the mutation and the variant were the molecular basis for his HVDRR. However, when we assessed the functionality of R370H [using the Sorting Intolerant from Tolerant (SIFT) web-based tool (http://sift.jcvi.org) and the Polymorphism Phenotyping 2 (PolyPhen2) tool (http://genetics.bwh.harvard.edu/pph2)] by homology modeling and threading, R370H was described as “tolerated” and “benign,” respectively. Furthermore, considering that rickets was dominantly inherited in this family (Fig. 1C), and that the patient showed a clinical course similar to that shown by his father, who had an identical heterozygous Q400LfsX7 mutation, we hypothesized that this mutation was the more likely molecular cause of his HVDRR. Indeed, the function of R370H-VDR was almost identical to wild-type VDR, including its transcriptional activity, ligand binding, and interactions with RXR, cofactors, and VDRE (Fig. 2, Fig. 3). Conversely, transcriptional activity and ligand binding ability of Q400LfsX7-VDR were completely impaired (Fig. 2A and C). More importantly, the mutant had a dominant-negative effect on wild-type-VDR (Fig. 2B). Thus, we concluded that the heterozygous Q400LfsX7 mutation caused the dominantly inherited HVDRR in this family through its dominant-negative effect on wild-type-VDR.

To our knowledge, this is the second reported pedigree of dominantly inherited HVDRR. The mutation detected in the first reported family (E420A) has been reported to show a dominant-negative effect (Malloy et al., 2011). In this study, we found that our Q400LfsX7 mutant differed from that in the previous report in terms of several functional properties. First, ligand binding was completely abolished in Q400LfsX7 (Fig. 2C), but remained in the E420A mutant. Second, Q400LfsX7 did not interact with RXR (Fig. 3A), while E420A could bind with RXR (Malloy et al., 2011). Third and importantly, our mutant strongly interacted with NCoR (Fig. 3C). Fourth, we demonstrated that Q400LfsX7 could interact with VDRE (Fig. 3D). These findings suggested that the dominant-negative effect of Q400LfsX7 was caused by a constitutive interaction with NCoR that may interact with VDRE to inhibit the activity of liganded wild-type-VDR. It is interesting that a similar molecular mechanism was postulated in the syndrome of resistance to thyroid hormone (RTH) caused by mutations in the TR α and β genes (*THRA* and *THRB*, respectively) (Safer et al., 1998, Liu et al., 1998, Fozzatti et al., 2011, Fozzatti et al., 2013, Bochukova et al., 2012). In contrast to HVDRR, RTH is usually an autosomal dominant disease. This dominant inheritance had been attributed to the dominant-negative effect of mutant *THRA* or *THRB* by failing to dissociate with NCoR (Safer et al., 1998, Fozzatti et al., 2011, Bochukova et al., 2012, Refetoff et al., 1993, Jameson, 1994). Most interestingly, the THRA E403X mutant lacks helix H12 and has similarly enhanced corepressor interaction and absent ligand binding through its loss of amino acids critical for hormone binding and coactivator recruitment, as shown by crystallographic modeling (Bochukova et al., 2012). We speculated that Q400LfsX7-VDR also caused an inability to release a corepressor by exposing a hydrophobic cleft on the receptor surface through the loss of helix H12. These findings suggested that HVDRR may be inherited as a dominant trait if the mutant has a constitutive corepressor interaction causing a dominant-negative effect, similar to RTH defects.

It is notable that our patient did not have alopecia. Cumulative data indicate that functional VDR is required for hair growth, and that alopecia is unrelated to the calcium or metabolic abnormalities that cause rickets (Malloy and Feldman, 2011). In addition, previous data indicate that VDR mutations cause defects in DNA binding, and that RXR heterodimerization or the absence of VDR causes alopecia, and mutations that alter VDR affinity for 1,25(OH)_2_D_3_ or disrupt coactivator interactions do not cause alopecia (Malloy and Feldman, 2011). Our finding suggested that, although Q400LfsX7-VDR did not interact with RXR, the mutant did not affect its dominant-negative effect on wild-type-VDR for the regulation of the hair cycle. A possible explanation is that Q400LfsX7-VDR may not be overexpressed compared with wild-type-VDR in the hair follicle. Further research using this mutant is necessary to elucidate the role of VDR in hair growth and differentiation.

Our results suggested that some patients with vitamin D-deficient rickets may have a *VDR* mutation. Our patient had obvious rickets and short stature but did not have hypocalcemia. His symptoms were relatively mild when compared with the classic clinical pattern of HVDRR, and his clinical course that the medication could be stopped by 4 years of age was similar to that of vitamin D-deficient rickets. In fact, his father was treated with vitamin D under the misdiagnosis of vitamin D-deficient rickets. In addition, aside from bowlegs, his unexamined paternal grandfather was otherwise asymptomatic without medication. The resolution of vitamin D resistance in patients with HVDRR has been previously described, but it typically occurs around puberty (Tiosano et al., 2011). We consider that mild cases of HVDRR may be misdiagnosed as vitamin D deficiency or remain undiagnosed. Our results expand the concept of HVDRR and enrich our understanding of VDR function in the pathogenesis of HVDRR.

## 5 Conclusion

We identified a novel *VDR* mutation (Q400LfsX7) with a dominant-negative effect on wild-type VDR in a family with dominantly inherited HVDRR, and we clarified that the mutant interacted strongly with NCoR. These findings suggested a distinct inheritance of HVDRR that expanded our understanding of the condition. Further investigations and pedigree analyses are needed to reveal the mechanism underlying dominantly inherited HVDRR.

## Disclosure statement

The authors declare no conflicts of interest.
